# Learning curve in 20 years of brace treatment

**DOI:** 10.1186/1748-7161-9-S1-O25

**Published:** 2014-12-04

**Authors:** Franz Landauer

**Affiliations:** 1 University Clinic of Orthopedics, Salzburg, Austria

## Background

The author has more than 20 years of experience in the treatment of scoliosis

## Aim

Objectification of his own learning curve in brace treatment.

## Design

Retrospective case study.

## Methods

I compared a group of 100 outpatients treated 20 years ago with a further group of 100 outpatients treated recently. The brace indication depends on the SOSORT-criteria. Cobb angle was measured before treatment, primary correction and follow up >1 years after weaning of the brace. Compliance was estimated by our own compliance score [1]. All braces are made by one orthotist.

## Results

### First examination

At the initial presentation Cobb angle changed from 31° (range 20°-56°) to 27° (range 20°-46°) in cases Risser <II. The number of pretreated patient without any orthopedic examination rise up to 8% and nearby 10% did not accept bracing. Also the number of non idiopathic scoliosis rise with the availability of MRI up to 6%.

### Bracing

Primary correction progressed in the group of 20°-30° (58% to 72%), 30°-40° (47% to 56%) and 40°- 50° (32% to 41%).

20 years ago I ordered 23 hours as fulltime bracing, but now 20 hours per day is the maximum, but the differentiation between good (73%) and bad compliance (27%) did not change significantly.

### Follow up

The final results at follow up have improved in the last 20 years from 28° to 23°, but the spread is still large. The number of progression >50° decreased from 12% to 5%.

### Summary

Now scoliosis is diagnosed earlier, but the number of unskilled pretreatment and rejection of treatment increased. Our experience lowers the number of idiopathic scoliosis. We have learned to make bracing more effective in primary correction and at follow up despite of a lower wearing time per day. We could not influence compliance significantly.

## Conclusion

Patients increasingly influence therapeutic regime. Part time bracing becomes the main option in outpatient treatment. There is still potential to improve brace-management. In outpatient treatment “compliance of the staff” seems to be a problem. An experienced orthopedic technician leads to a good standard, but it lacks on comparison and competition.
